# EOLA1 protects lipopolysaccharide induced IL-6 production and apoptosis by regulation of MT2A in human umbilical vein endothelial cells

**DOI:** 10.1007/s11010-014-2110-7

**Published:** 2014-06-11

**Authors:** Yueming Liu, Hairong Liu, Wenhai Chen, Ting Yang, Wei Zhang

**Affiliations:** Burn and Plastic Surgery Department of the First Hospital of Chengdu Medical College, 278 Baoguang Road, Xindu District, Chengdu, 610500 China

**Keywords:** Inflammation, IL-6, Lipopolysaccharide, EOLA1, MT2a

## Abstract

Endothelial cell (EC) injury or dysfunction is believed to be mediated at least in part by lipopolysaccharide (LPS). Recent studies have shown that LPS induces apoptosis in different types of endothelium, including HUVEC. Previously we used EOLA1 (endothelial-overexpressed LPS-associated factor 1) cDNA as a bait and performed a yeast two-hybrid screening of a human liver cDNA library and identified metallothionein 2a (MT2a) as the associated protein. EOLA1 protein plays a role as a signal transduction factor. But the mechanism of EOLA1 mediated the protection of cell production of IL-6 and apopotosis in HUVEC is not known. MT2a is expressed in many kinds of cells and plays a role in inflammation. In this study, we demonstrated that LPS could induce EOLA1 expression in time-dependent and apparently contributed to the inhibition of IL-6 production and apoptosis induced by LPS treatment. We also found that deletion of EOLA1 promoted IL-6 production and apoptosis in the treatment of LPS in HUVEC. Furthermore, we demonstrated that MT2a was activated by LPS, and played a key role in LPS-induced IL-6 expression in HUVEC. We further provided the evidence that EOLA1 functioned as a negative regulator for LPS response by regulation of MT2a. These findings suggest that EOLA1 may have an important regulatory role during EC inflammatory responses.

## Introduction

Endothelial cell (EC) injury and/or dysfunction, a commonality among several key complications associated with septic shock, is believed to be mediated at least in part by lipopolysaccharide (LPS). LPS, a complex glycoprotein constituent of the outer cell wall of Gram-negative bacteria, has been implicated in endothelial injury that leads to septic shock and its associated syndromes [1–5]. Recent studies have shown that LPS induces apoptosis in different types of endothelium, including HUVEC and lung-derived normal human microvascular endothelial cells [6–9]. LPS administration has also been shown to cause apoptosis in B cells thymocytes, and lymphoid organs [10, 11]. Enhanced apoptotic cell death has also been shown in various tissues derived from patients who have died due to sepsis or multiorgan failure. LPS directly activates the vascular endothelium and elicits an array of EC responses, including an increase in the expression of specific adhesion molecules and inflammatory cytokines such as IL-1, IL-8, and MCP-1 (monocyte chemotactic protein-1), which in turn results in the selective recruitment of leukocytes to inflammatory foci [12, 13]. Previous studies have also reported that release of LPS into the circulation induces endothelial apoptosis in vivo and thus causes microvascular injury in numerous tissues, including lung, gut, and liver, during sepsis [1–3]. Enhanced apoptotic cell death has also been shown in various tissues derived from patients who have died due to sepsis or multiorgan failure. Apoptotic endothelial cells have also been detected in murine models of sepsis [4, 12].

EOLA1, named as endothelial-overexpressed LPS-associated factor 1 (also named as Cxorf40A), is 1404 bp long, encoding a 158aa, 17.8 kDa protein, mapped to chromosome Xq27.4 with 5 exons EOLA1 was mainly expressed in *E. coli* as insoluble inclusion bodies. The protein content in the primary extracted inclusion bodies accounted for over 75 %, and it accounted for more than 90 % after chromatography and renaturation [13]. It is expressed primarily in heart, skeletal muscle, kidney, liver and placenta. Relatively high level of expression in spleen, colon and small intestine and also cancer cell lines. Almost no expression in brain, thymus, lung and peripheral blood leukocytes. Previous report showed that EOLA1 protein is localized in the nucleus and the matrix of ECV304 cells, and it plays a role as a signal transduction factor [14, 15]. EOLA1 could inhibit the proliferation of human umbilical vein EC line ECV304 [16, 17].

Metallothioneins (Mts) are a family of proteins with a high affinity to certain metal ions such as zinc and cadmium. Mts proteins are expressed in multiple organs and exist in several isoforms subdivided in four groups Mt1, Mt2, Mt3 and Mt4. Mts may have a role in the regulation of zinc and copper homeostasis and act as potent antioxidants against oxidative damage [18, 19]. MT2a is one of the famliy and express in many kinds of cells such as 3T3-L1 adipocytes, cancer cells [20–22]. A significant association between rs28366003 genotype and MT2a expression level is found in prostate cancer patients and other cells. MT2a has various functions including involving in insulin resistance in fat cells; predicting poor outcome in non-small cell lung cancer [23–28]. EOLA1 and MT2a may have an important role of cell protection in inflammation reaction.

To investigate the role of EOLA1 in LPS induced IL-6 production and apoptosis, this study was designed to examine their possible contribution to LPS-stimulated IL-6 expression in HUVEC. We demonstrated, for the first time, that EOLA1 expression was induced by LPS in HUVEC, and apparently contributed to the inhibition of IL-6 induction by LPS treatment. Furthermore, we found that EOLA1 inhibited LPS-induced IL-6 expression and apoptosis in HUVEC by MT2a. The data suggest that EOLA1 may have an important regulatory role during HUVEC-associated inflammatory responses.

## Materials and methods

### Cell culture

HUVEC cell line was purchased from ATCC (Manassas, VA, USA). Cells were grown at 37 °C in 5 % CO2 in endothelial growth medium (EGM-2-MV) containing 2 % FBS, 12 μg/ml bovine brain extract, 10 ng/ml human recombinant epidermal growth factor, 1 μg/ml hydrocortisone, GA-1000 (gentamicin and amphotericin B, 1 μg/ml), according to the recommendations of the supplier.

### siRNA treatment

Knockdown of EOLA1 and MT2a was accomplished using siRNA (synthesized by Genepharma, Shanghai, China). EOLA1 siRNA target sequence was: 5′-AAGTGGAAGAGTGTTTCCTCC-3′ and MT2a siRNA target sequence was: 5′-AAGTGCAGCTGCTGCGCCTGA-3′. Approximately 2 × 10^5^ cells were seeded per well of a 6-well tissue culture dish the day before transfection. Transfection was performed according to the manufacturer’s instructions using lipofectamine-2000 reagent and 100nM siRNA. Efficient knockdown was checked three day post-transfection of siRNA by RT-PCR and Western blotting.

### Cytokines assay

The cells were homogenized in PBS (1:2, w/v) containing 1 % protease inhibitors and then centrifuged at 12,000 × *g* for 15 min at 4 °C. The supernatants were analyzed for IL-6 using ELISA kit (Roche, USA) according to the manufacturer’s instructions.

### RNA isolation and real-time RT-PCR

Total RNA, following the manufacturer’s instructions, was isolated from the cells using Trizol reagent (Invitrogen). Briefly, the cells were lysed in TRIzol and then mixed with chloroform. The lysate was centrifuged to separate RNA, DNA and protein, total RNA recovered, precipitated with isopropanol, washed in 75 % ethanol to remove impurities before dissolved in water. After that, 2 μg of RNA was taken and treated with DNase to remove contaminating DNA prior to the reverse transcription to cDNA. To measure mRNA expression, real-time RT-PCR was performed using a sequence detector (ABI-Prism, Applied Biosystems, USA). The primers are: EOLA1 upstream:5′-GCTCGAATTCATGAAGAAGTTTGGCTGCCTCTC-3′,downstream:5′-AGCAGGATCCTCTCTTCATGCCCCAAAG-3′. MT2a: upstream:5′-CCAATAGATCTGCCACCA-3′,downstream:5′-ATTGGGGTACCGTGGCGCA-3′. The relative expression levels were calculated by comparing Ct values of the samples with those of the reference, all data normalized to the internal control GAPDH.

### Construction of EOLA1 vector, lentivirus production and infection

The full-length human EOLA1 cDNA sequence was amplified by PCR of EOLA1 vector (OriGene, MD, USA) and subcloned into the lentivirus vector (Invitrogen) for the generation of EOLA1 vector. The construct was verified by sequencing. The lentivirus was produced according to the manufacturer’s instructions: DNA–Lipofectamine 2000 complexes were prepared containing 3 mg of lentivirus vector expression plasmid DNA and 9 mg of the ViraPower Packaging Mix in 1.5 ml of Opti-MEM I medium without serum; the packaging cell 293FT was trypsinized and counted and the cells were resuspended at a density of 10^6^ cells/ml in growth medium containing serum; the DNA–Lipofectamine 2000 complexes were added to a 10-cm culture plate containing 5 ml of growth medium with serum; 5 ml 293FT cells were added to the plate and mixed gently by rocking. On the next day, the media containing the DNA–Lipofectamine 2000 complexes were removed and replaced with complete culture medium, and virus-containing supernatants 48 h post-transfection were harvested and centrifuged at 3,000 rpm for 5 min at 48 °C to pellet debris, and the virus was concentrated at 20,000 rpm and stored at −70 °C, lentiviral vectors were titered in the cells based on the protocols.

### Determination of apoptosis by flow cytometry

For apoptosis assay, the AnnexinnV straining was quantified by flow cytometric. The cells were plated in a 6-well plate, transfected with the indicated plasmid or siRNA, at 24 h later, the complete growth medium were changed to growth medium without serum. At another 24 h later, the cells were collected, washed in cold PBS twice and resuspended in 1× binding buffer at a concentration of 1 × 10^6^ cells/ml. After that, the cells in 100 µl solution were transferred to a 5 ml culture tube, with 5 µl Annexin V-FITC and 5 µl PI (BD Biosciences) added, and gently vortexed and incubated for 15 min at RT in the dark. And finally, 400 µl 1× binding buffer was added to each tube to be analyzed by flow cytometry within 1 h.

### Western blotting

The cells were scraped from the dishes, cellular protein extracts prepared by homogenization in an ice-cold lysis buffer and their lysates obtained by centrifugation at 12,000 × *g* for 20 min, and the total protein concentration determined using Lowry method. Equal amounts of protein, separated by SDS-PAGE, were electrophoretically transferred to a PVDF membrane at 320 mA for 2 h at a low temperature and the membrane was blocked with 5 % fat-free milk with 0.05 % Tween 20 in PBS. Subsequently, the membrane was probed with the primary antibodies. The blots were washed in PBST and then incubated in anti-mouse IgG or anti-rabbit IgG secondary antibody for about 3 h at RT. Washed in PBST (×3, 10 min each wash), the proteins were finally visualized using ECL based on the manufacturer’s instructions.

### Statistical analysis

Each experiment was repeated at least three times, Student’s *t* tests performed to determine the statistical significance for the assays of ELISA and real time RT-PCR, error bars representing ± SE.

## Results

### LPS induces IL-6 production and EOLA1 expression in HUVEC

**Fig. 1:**
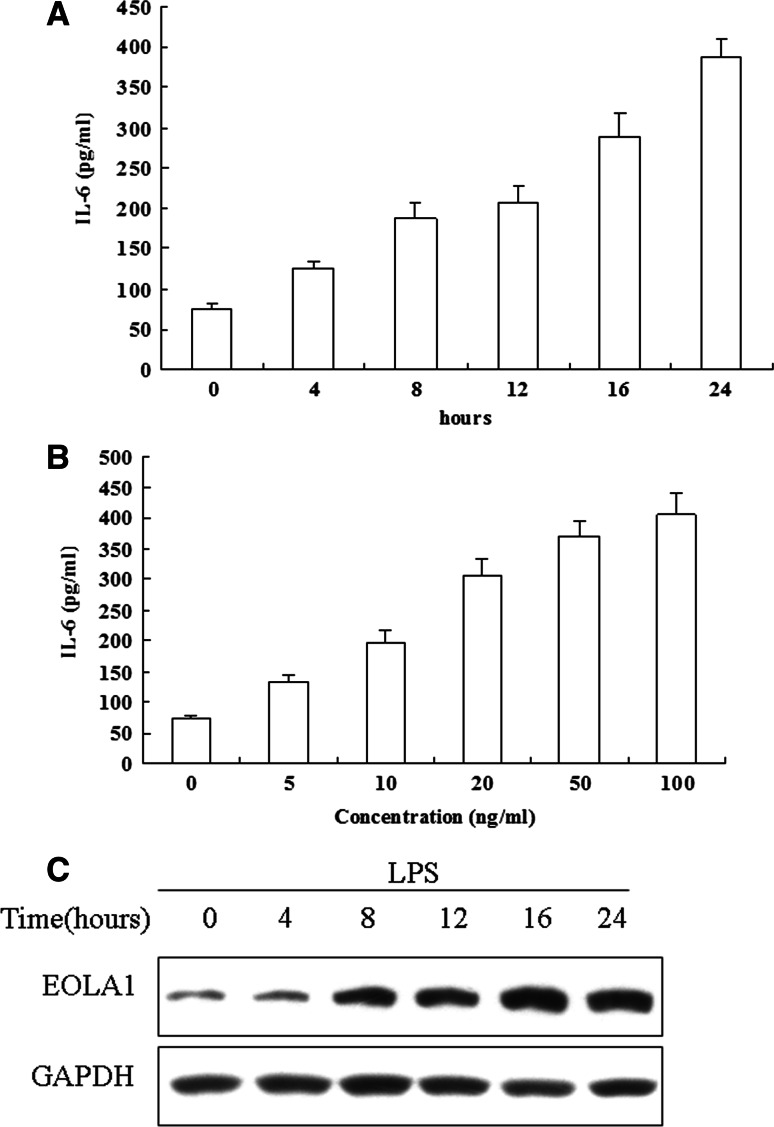
LPS induces IL-6 production and EOLA1 in human umbilical vein endothelial cells (HUVEC). **a** HUVEC were treated with LPS (100 ng/ml) for various periods of time 0, 4, 8, 16 h and the supernatants were collected and assayed for IL-6 production by ELISA. **b** HUVEC were treated with various doses of LPS 0, 1, 10, 50 and 100 ng/ml for 16 h and he supernatants were collected and assayed for IL-6 production by ELISA. Data represent the mean ± S.D of three independent experiments. **c** LPS induces EOLA1 expression in HUVEC. Cells were stimulated with LPS (100 ng/ml) for the indicated periods of time. The lysates were analyzed by Western blotting with antibodies to EOLA1. Data show one representative experiment out of three independent experiments

To examine the modulation of IL-6 secretion from endothelial cells on LPS treatment, HUVEC were grown to 80 % confluence. These cells were stimulated with LPS for the indicated periods. IL-6 concentrations in the clarified culture supernatants were then measured by ELISA. Exposure of HUVEC to LPS (100 ng/ml) resulted in a significantly greater increase in IL-6 production compared to HUVEC (Fig. 1a). Up-regulation of IL-6 in cells was time-dependent (Fig. 1a) and dose-dependent (Fig. 1b).

Previous report showed that EOLA1 protein is localized in the nucleus and the matrix of ECV304 cells, and it plays a role as a signal transduction factor. EOLA1 could inhibit the proliferation of human umbilical vein EC line ECV304. To investigate the signaling processes involved in LPS-induced IL-6 production, HUVEC was stimulated with 100 ng/ml LPS for different lengths of time and showed that EOLA1 was decreased in time-dependent (Fig. 1c).

### Deletion of EOLA1 increased the expression of IL6 and apoptosis induced by LPS in HUVEC

**Fig. 2:**
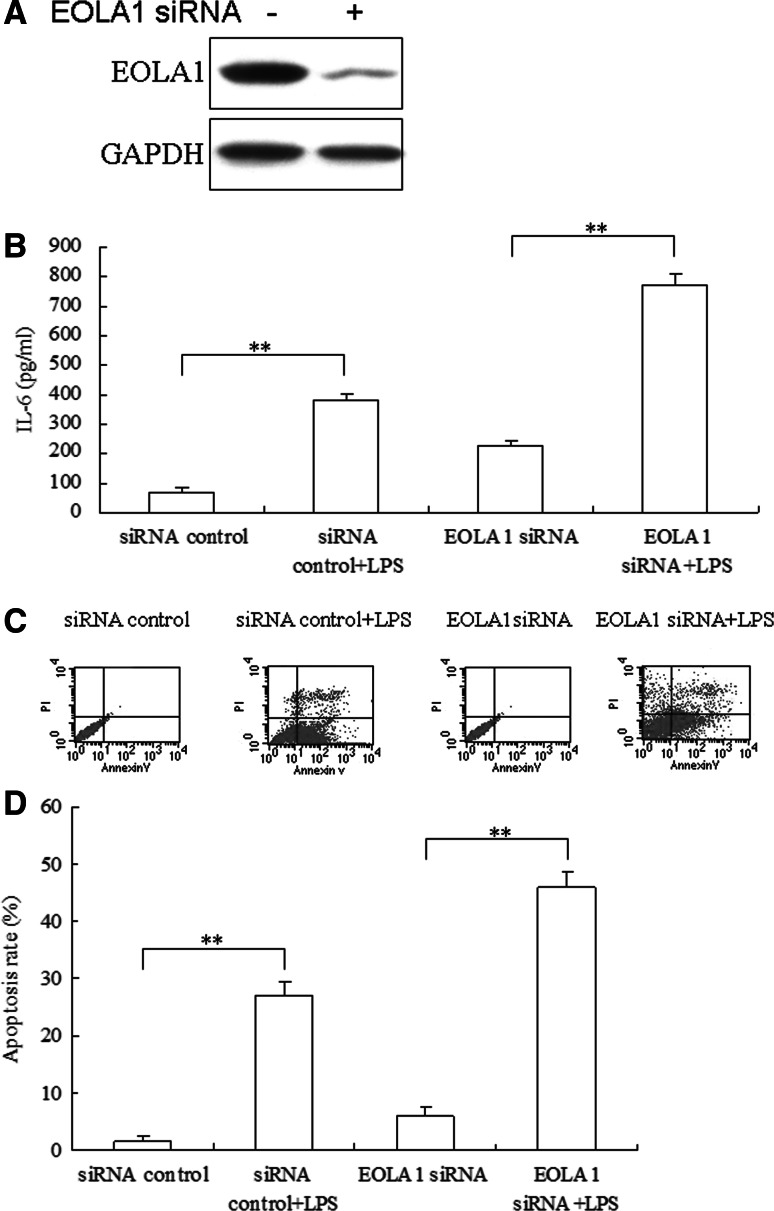
Inhibition of EOLA1 with siRNA inhibits LPS-induced IL-6 production and induces apotosis in HUVEC. **a** HMVEC were transfected with control or EOLA1 siRNA (200 nM) for 48 h at 37 °C. The cells were lysed and analyzed by Western blotting with EOLA1 antibody. **b** HMVEC were transfected with control or EOLA1 siRNA (200 nM) for 24 h at 37 °C and then cultured with or without LPS (100 ng/ml) and the concentration of IL-6 in the culture supernatants was determined 16 h after stimulation. Data represent the mean ± S.D of three independent experiments. (C) HMVEC cells pre-treated with EOLA1 siRNA were stimulated with LPS (100 ng/ml) for 16 h. The supernatants were then tested for apoptosis. (**P < 0.01, *P < 0.05)

To further research the role of EOLA1 in HUVEC, the cells were transfected with EOLA1 siRNA to observe expression of IL-6. EOLA1 was down-regulated in the HUVEC with siRNA (Fig. 2a). The cells with depletion of EOLA1 were treated with LPS (100 ng/ml) at the point of 48 h post-transfection and IL6 was detected by ELISA assay 6 h after LPS induced. The results showed that LPS could induce IL6 expression, knocking down of EOLA1 increased the expression of IL6 of HUVEC cells induced by LPS in HUVEC cells (Fig. 2b). The cells with depletion of EOLA1 were treated with LPS (100 ng/ml) at the point of 48 h post-transfection and IL6 was detected by ELISA assay 24 h after LPS induced. The results showed that knocking down of EOLA1 increased apoptosis of HUVEC cells induced by LPS in HUVEC cells (Fig. 2c). These results meant that EOLA1 could inhibit the expression of IL-6 and apoptosis in HUVEC cells induced by LPS.

### Overexpression of EOLA1 inhibited the expression of IL6 and apoptosis induced by LPS in HUVEC

**Fig. 3:**
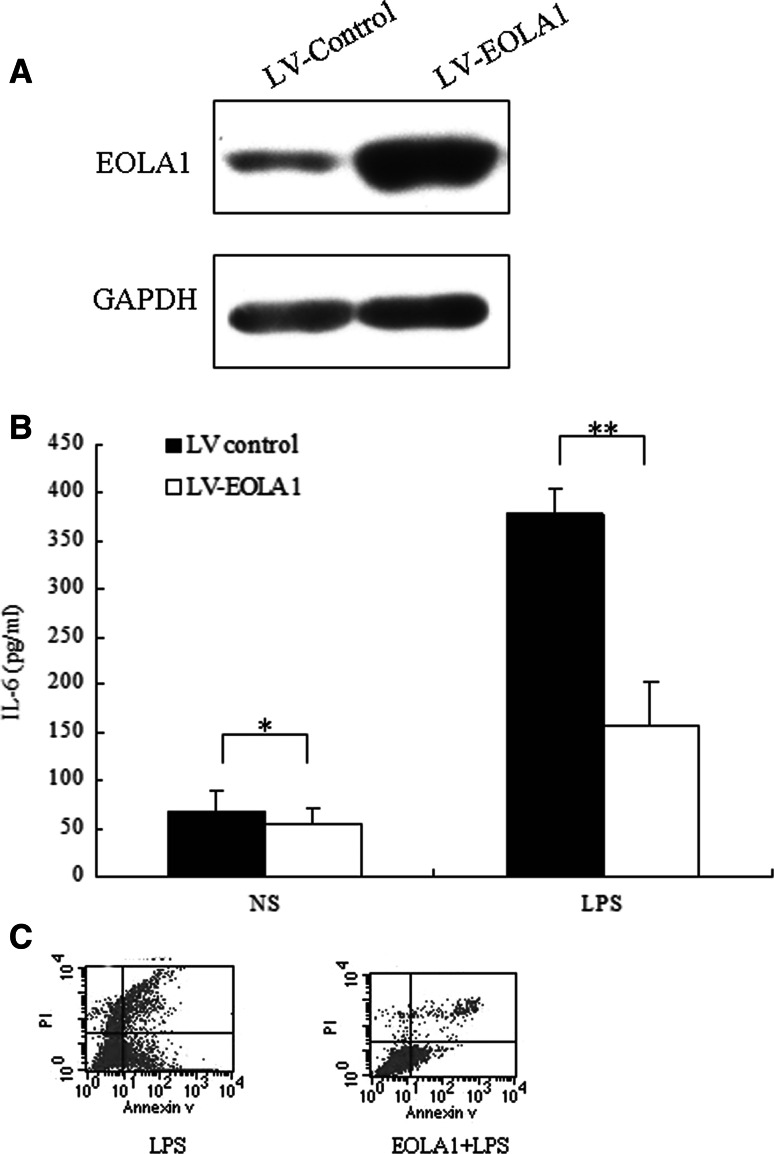
Transduction of HMVECs with EOLA1 prevents LPS induced IL-6 production and inhibits apoptosis. HMVEC cells were transduced with lentivirus mediated EOLA1. **a** Overexpression of the EOLA1 was demonstrated by Western blot analysis with anti-EOLA1 antibody 48 h after transduction. **b** Transduced cells were tested for their ability to produce IL-6 upon LPS stimulation (100 ng/ml) using a commercial ELISA kit. **c** Apoptosis was tested by flow cytometry. Data represent the mean ± S.D of three independent experiments. (**P < 0.01, *P < 0.05)

To further research the role of EOLA1 in HUVEC, the cells were transfected with lentivirus mediated EOLA1 to observe expression of IL-6. EOLA1 was overexpressed in the HUVEC (Fig. 3a). The cells with depletion of EOLA1 were treated with LPS (100 ng/ml) at the point of 48 h post-infection and IL6 was detected by ELISA assay 6 h after LPS induced. The results showed that LPS could induce IL6 expression, knocking down of EOLA1 increased the expression of IL6 of HUVEC cells induced by LPS in HUVEC cells (Fig. 3b). The cells with depletion of EOLA1 were treated with LPS (100 ng/ml) at the point of 48 h infection and IL6 was detected by ELISA assay 24 h after LPS induced. The results showed that EOLA1 inhibited apoptosis of HUVEC cells induced by LPS in HUVEC cells (Fig. 3c). These results verified that EOLA1 could inhibit the expression of IL-6 and apoptosis in HUVEC cells induced by LPS. The data suggested that EOLA1 might play a role in stress such as inflammation reaction.

### EOLA1 regulated IL-6 production through MT2a

**Fig. 4:**
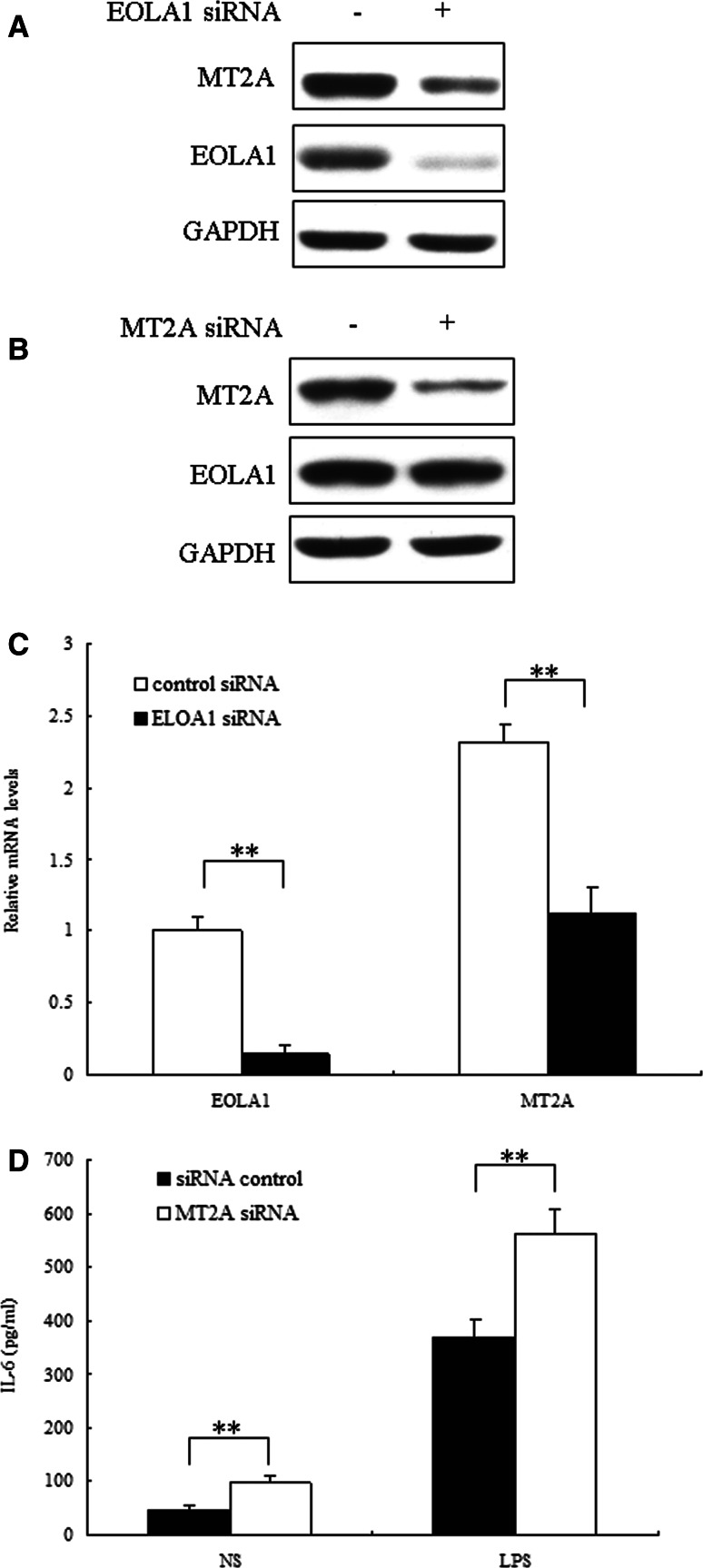
EOLA1 mediates LPS induced IL-6 production and inhibits apoptosis by MT2a. **a** HUVEC was transfected with EOLA1 siRNA or control siRNA, and the cells were lysed and analyzed by Western blotting with anti-MT2a antibody. **b** HUVEC was transfected with MT2a siRNA or control siRNA, and total RNA was isolated and analyzed by real tim RT-PCR. **c** HUVEC was transfected with MT2a siRNA or control siRNA, and the cells were lysed and analyzed by Western blotting with anti-MT2a antibody. **d** HMVEC was transfected with MT2a siRNA or control siRNA and then stimulated with LPS (100 ng/ml)for 16 h. IL-6 production was assayed by ELISA. (**P < 0.01, *P < 0.05)

To investigate the mechanism by which EOLA1 mediates IL-6 production, we focused on MT2a. Our previous work showed that MT2a was an associated protein may be related to EOLA1. To explore whether EOLA1 regulates the expression of MT2a, MT2a protein and mRNA were measured by Western Blot and Real time PCR after cells were transfected with EOLA1 siRNA. The results showed that knocking down of EOLA1 could inhibit the expression of MT2a significantly (Fig. 4a, b). This result indicated that EOLA1 regulated the expression of MT2a in HUVEC cells. To study whether MT2a involves in the IL-6 production by LPS, the cells with depletion of MT2a were treated with LPS (100 ng/ml) at the point of 48 h post-transfection and IL6 was detected by ELISA assay 6 h after LPS induced. The results showed that knocking down of MT2a increased the expression of IL6 of HUVEC cells induced by LPS in HUVEC cells (Fig. 4c). The cells with depletion of EOLA1 were treated with LPS (100 ng/ml) at the point of 48 h post-transfection and apoptosis was detected by flow cytometry after LPS induced. The results showed that knocking down of MT2a increased apoptosis of HUVEC cells induced by LPS in HUVEC cells (Fig. 4d). These results meant that EOLA1 could inhibit the expression of IL-6 and apoptosis in HUVEC cells induced by LPS through MT2a.

## Discussion

A great mount of research results show that vascular EC damage is the important factors led to shock and multiorgan injury. Overactivation of endothelial cells can result in uncontrolled inflammation. It is known that ischemia, hypoxia, and endotoxin can make endothelial cells in the stress, however, the mechanism is not clear.

The bacterial endotoxin, LPS stimulates endothelial cells to express chemokines that initiate the activation and recruitment of circulating leukocytes at inflammatory tissue sites [29, 30]. But the effects of LPS on cytokine production in endothelial cells are well elucidated, the mechanism involved in this process has not been characterized in detail. Hence we questioned whether EOLA1 has a role in LPS-induced production of IL-6, attempting to elucidate the molecular mechanism involved in this pathway. Here, we demonstrate EOLA1 mediates LPS-induced IL-6 production. Our experiments demonstrate that LPS increases EOLA1 in microvascular endothelial cells. The involvement of EOLA1 in LPS-induced IL-6 production was confirmed using multiple strategies, namely LV-expressing EOLA1 and EOLA1-specific siRNA.

Previous study showed that EOLA1 expression is associated with vascular endothelial cells stimulated by LPS and may involved in the process of activation of endothelial cells and its damage [15, 31]. EOLA1 could protect endothelial cells inflammation and LPS could induce EOLA1 expression. EOLA1 is relatively novel gene, which was discovered in recent years and its function is not clear. Previous research indicated that it is expressed in various of cells which indicated it may play important role in the cells. Our data suggested that EOLA1 might induce apoptosis resistance and protect the endothelial cells in the stress. The results were consistent with the previous report. So it is a protecting molecule of cells. These indicates that EOLA1 plays important roles in cell injury, wounding and stress reaction.

Based on EOLA1’s secondary structure, EOLA1 is predicted to have the phosphorylated sites of tyrosine kinase and protein kinase C and HTH motif, which might make it has the ability of signal transduction. EOLA1 may play an important role in activate endothelial cells induced by LPS. EOLA1 is strongly expressed in many tissues such as heart, bone, kidney, liver and placenta weakly expressed in colon, small intestine and spleen, however almost no expression in brain, thymus, lung, and peripheral white blood cells. All of these indicates that EOLA1 is preferred to expressing in the tissues with rich blood. MT2a is an important stressful protection protein and induced by LPS, which involves in cell proliferation, differentiation. The relationship of EOLA1 and MT2a and their sublocation in the cells suggested that EOLA1 might be transcription factor associated with cell activation stimulated by LPS.

Taken together, we reported here that EOLA1 in HUVEC dramatically decreased inflammation factor IL-6 production and apoptosis induced by LPS treatment. Our finding is the first demonstration that EOLA1 expression has a functional role in inflammation of HUVEC. Our studies indicate that EOLA1 may function as an important regulator in protection of HUVEC injury in inflammatory diseases.

## References

[1] Miller AM, Horiguchi N, Jeong WI, Radaeva S, Gao B (2011). Molecular mechanisms of alcoholic liver disease: innate immunity and cytokines. Alcohol Clin Exp Res.

[2] Dauphinee SM, Karsan A (2006). Lipopolysaccharide signaling in endothelial cells. Lab Invest.

[3] Bannerman DD, Goldblum SE (2003). Mechanisms of bacterial lipopolysaccharide-induced endothelial apoptosis. Am J Physiol Lung Cell Mol Physiol.

[4] Wort SJ, Evans TW (1999). The role of the endothelium in modulating vascular control in sepsis and related conditions. Br Med Bull.

[5] Koide N, Abe K, Narita K, Kato Y, Sugiyama T, Jiang GZ, Yokochi T (1996). Apoptotic cell death of vascular endothelial cells and renal tubular cells in the generalized Shwartzman reaction. FEMS Immunol Med Microbiol.

[6] Hotchkiss RS, Swanson PE, Freeman BD, Tinsley KW, Cobb JP, Matuschak GM, Buchman TG, Karl IE (1999). Apoptotic cell death in patients with sepsis, shock, and multiple organ dysfunction. Crit Care Med.

[7] Berman RS, Frew JD, Martin W (1993). Endotoxin-induced arterial endothelial barrier dysfunction assessed by an in vitro model. Br J Pharmacol.

[8] Trepels T, Zeiher AM, Fichtlscherer S (2006). The endothelium and inflammation. Endothelium.

[9] Wang Y, Chen H, Li H, Zhang J, Gao Y (2013). Effect of angiopoietin-like protein 4 on rat pulmonary microvascular endothelial cells exposed to LPS. Int J Mol Med.

[10] Hu G, Liu J, Zhen YZ, Wei J, Qiao Y, Lin YJ, Tu P (2013). Rhein inhibits the expression of vascular cell adhesion molecule 1 in human umbilical vein endothelial cells with or without lipopolysaccharide stimulation. Am J Chin Med.

[11] Sakurai A, Kinoshita K, Furukawa M, Noda A, Yamaguchi J, Kogawa R, Tanjoh K (2012). Implication for Long-Term Hypothermia on Degradation of Interleukin-8 mRNA in Endothelial Cells Stimulated with Lipopolysaccharides. Ther Hypothermia Temp Manag.

[12] Echeverría C, Montorfano I, Sarmiento D, Becerra A, Nuñez-Villena F, Figueroa XF, Cabello-Verrugio C, Elorza AA, Riedel C, Simon F (2013). Lipopolysaccharide induces a fibrotic-like phenotype in endothelial cells. J Cell Mol Med.

[13] Yokochi T, Kato Y, Sugiyama T, Koide N, Morikawa A, Jiang GZ, Kawai M, Yoshida T, Fukada M, Takahashi K (1996). Lipopolysaccharide induces apoptotic cell death of B memory cells and regulates B cell memory in antigen-nonspecific manner. FEMS Immunol Med Microbiol.

[14] Kato Y, Morikawa A, Sugiyama T (1997). Augmentation of lipopolysaccharide-induced thymocyte apoptosis by interferon-gamma. Cell Immunol.

[15] Cai Z, Liang ZW, Luo XD, Yang ZC, Sun RJ, Su YY (2005). Purification of human endothelial overexpressed lipopolysaccharide-associated factor 1 protein. Zhonghua Shao Shang Za Zhi.

[16] Luo M, Liang ZW, Yang ZC, Luo XD (2010). Subcellular localization of human endothelial-overexpressed lipopolysaccharide-associated factor 1 protein. Zhonghua Shao Shang Za Zhi.

[17] Liu YM, Liu HR, Cai Z, Ma B, Liu YL, Zhang W (2010). Preparation of polyclonal antibody of human endothelial-overexpressed lipopolysaccharide-associated factor 1. Zhonghua Shao Shang Za Zhi.

[18] Liang ZW, Yang ZC, Chen J, Luo XF, Wang XM (2007). The effect of inhibiting EOLA1 expression in ECV304 cells. Zhonghua Yi Xue Yi Chuan Xue Za Zhi.

[19] Liang ZW, Yang ZC, Liu YM, Chen Y, Luo XD (2005). Identification and characterization of a novel gene EOLA1 stimulating ECV304 cell proliferation. Zhonghua Yi Xue Yi Chuan Xue Za Zhi.

[20] Kim HG, Kim JY, Han EH, Hwang YP, Choi JH, Park BH, Jeong HG (2011). Metallothionein-2A overexpression increases the expression of matrix metalloproteinase-9 and invasion of breast cancer cells. FEBS Lett.

[21] Martinho A, Gonçalves I, Cardoso I, Almeida MR, Quintela T, Saraiva MJ, Santos CR (2010). Human metallothioneins 2 and 3 differentially affect amyloid-beta binding by transthyretin. FEBS J.

[22] Wesselkamper SC, McDowell SA, Medvedovic M, Dalton TP, Deshmukh HS, Sartor MA, Case LM, Henning LN, Borchers MT, Tomlinson CR, Prows DR, Leikauf GD (2006). The role of metallothionein in the pathogenesis of acute lung injury. Am J Respir Cell Mol Biol.

[23] Yamasaki M, Nomura T, Sato F, Mimata H (2007). Metallothionein is up-regulated under hypoxia and promotes the survival of human prostate cancer cells. Oncol Rep.

[24] Reinecke F, Levanets O, Olivier Y, Louw R, Semete B, Grobler A, Hidalgo J, Smeitink J, Olckers A, Van der Westhuizen FH (2006). Metallothionein isoform 2A expression is inducible and protects against ROS-mediated cell death in rotenone-treated HeLa cells. Biochem J.

[25] Rao PS, Jaggi M, Smith DJ, Hemstreet GP, Balaji KC (2003). Metallothionein 2a interacts with the kinase domain of PKCmu in prostate cancer. Biochem Biophys Res Commun.

[26] Werynska B, Pula B, Muszczynska-Bernhard B, Gomulkiewicz A, Piotrowska A, Prus R, Podhorska-Okolow M, Jankowska R, Dziegiel P (2013). Metallothionein 1F and 2A overexpression predicts poor outcome of non-small cell lung cancer patients. Exp Mol Pathol.

[27] Haynes V, Connor T, Tchernof A, Vidal H, Dubois S (2013). Metallothionein 2a gene expression is increased in subcutaneous adipose tissue of type 2 diabetic patients. Mol Genet Metab.

[28] Wojnar A, Pula B, Piotrowska A, Jethon A, Kujawa K, Kobierzycki C, Rys J, Podhorska-Okolow M, Dziegiel P (2011). Correlation of intensity of MT-I/II expression with Ki-67 and MCM-2 proteins in invasive ductal breast carcinoma. Anticancer Res.

[29] Anand AR, Bradley R, Ganju RK (2009). LPS-induced MCP-1 expression in human microvascular endothelial cells is mediated by the tyrosine kinase, Pyk2 via the p38 MAPK/NF-kappaB-dependent pathway. Mol Immunol.

[30] Munshi N, Fernandis AZ, Cherla RP, Park IW, Ganju RK (2002). Lipopolysaccharide-induced apoptosis of endothelial cells and its inhibition by vascular endothelial growth factor. J Immunol.

[31] Liang Z, Yang Z (2004). Identification and characterization of a novel gene EOLA1 stimulating ECV304 cell proliferation. Biochem Biophys Res Commun.

